# Evolution of cichlid vision via *trans*-regulatory divergence

**DOI:** 10.1186/1471-2148-12-251

**Published:** 2012-12-26

**Authors:** Kelly E O’Quin, Jane E Schulte, Zil Patel, Nadia Kahn, Zan Naseer, Helena Wang, Matthew A Conte, Karen L Carleton

**Affiliations:** 1 Department of Biology, University of Maryland, College Park, MD, 20742, USA

## Abstract

**Background:**

Phenotypic evolution may occur through mutations that affect either the structure or expression of protein-coding genes**.** Although the evolution of color vision has historically been attributed to structural mutations within the opsin genes, recent research has shown that opsin regulatory mutations can also tune photoreceptor sensitivity and color vision. Visual sensitivity in African cichlid fishes varies as a result of the differential expression of seven opsin genes**.** We crossed cichlid species that express different opsin gene sets and scanned their genome for expression Quantitative Trait Loci (eQTL) responsible for these differences. Our results shed light on the role that different structural, *cis*-, and *trans*-regulatory mutations play in the evolution of color vision.

**Results:**

We identified 11 eQTL that contribute to the divergent expression of five opsin genes. On three linkage groups, several eQTL formed regulatory “hotspots” associated with the expression of multiple opsins. Importantly, however, the majority of the eQTL we identified (8/11 or 73%) occur on linkage groups located *trans* to the opsin genes, suggesting that cichlid color vision has evolved primarily via *trans*-regulatory divergence. By modeling the impact of just two of these *trans*-regulatory eQTL, we show that opsin regulatory mutations can alter cichlid photoreceptor sensitivity and color vision at least as much as opsin structural mutations can.

**Conclusions:**

Combined with previous work, we demonstrate that the evolution of cichlid color vision results from the interplay of structural, *cis*-, and especially *trans*-regulatory loci. Although there are numerous examples of structural and *cis*-regulatory mutations that contribute to phenotypic evolution, our results suggest that *trans*-regulatory mutations could contribute to phenotypic divergence more commonly than previously expected, especially in systems like color vision, where compensatory changes in the expression of multiple genes are required in order to produce functional phenotypes.

## Background

Phenotypic evolution can result from mutations that affect either the structure or expression of protein-coding genes. The evolution of vertebrate color vision has primarily been attributed to structural mutations within the opsin genes [1], a group of G-protein-coupled receptors expressed within the light-sensitive photoreceptor cells of the retina [2]. These structural mutations include protein-coding substitutions that alter the polarity of amino acids within the functional retinal-binding region of the opsin protein, causing the opsins and the photoreceptors they are expressed within to absorb light at longer or shorter wavelengths, thereby altering color vision (e.g., [3,4]). But recent research has shown that photoreceptor sensitivity can also evolve through regulatory mutations that affect opsin expression [5-7], although the genetic basis of these regulatory changes is largely unknown. Since a complex network of both *cis*- and *trans-*regulatory factors controls vertebrate opsin expression [8-10], quantitative genetic studies of opsin gene regulation have the potential to clarify the role that different structural, *cis*-, and *trans*-regulatory mutations play in the evolution of color vision.

African cichlid fishes have undergone a dramatic adaptive radiation that involves many traits, including color vision [11]. In some cases, closely related cichlids differ in the maximum sensitivity of their photoreceptors by as much as 100 nm [12]. The primary genetic mechanism responsible for this diversity is the differential regulation of seven opsin genes, although structural mutations also play a role [13,14]. These seven opsins are maximally sensitive to different wavelengths of light and include *SWS1* (ultra-violet-sensitive), *SWS2B* (violet-sensitive), *SWS2A* (blue-sensitive), *RH2B* (blue-green-sensitive), *RH2A-α* and -*β* (green-sensitive), and *LWS* (red-sensitive) [15]). The expression of these genes varies qualitatively among cichlids, resulting in groups of species with distinct “visual palettes”, or sets of photoreceptors broadly sensitive to short-, middle-, or long-wavelength light [13,16]. Within these groups, cichlids also exhibit quantitative variation in the expression of pairs of opsins, including the *SWS2B/SWS2A* opsin pair and the *RH2A/LWS* pair, where a reduction in the expression of one gene is compensated by an increase in the expression of the other. Such qualitative and quantitative changes in opsin expression may serve to fine-tune cichlid vision in response to specific prey items, changes in the ambient light environment, or conspecific signals [13,16].

The genetic factors responsible for interspecific variation in cichlid opsin expression and photoreceptor sensitivity are currently unknown. In two previous studies [17,18], we used (a) a scan of non-coding DNA located near the opsins and (b) a genetic cross of two Lake Malawi cichlids that express different visual palettes to determine that both *cis*- and *trans*-regulatory factors contribute to the evolution of cichlid color vision, although we could not resolve the location or relative importance of these loci. Here, we expand on our previous studies by sampling a larger number of F_2_ progeny from the hybrid cross used in Carleton et al. [17]. We perform a genome scan of > 500 sequenced restriction-site associated DNA (RAD-seq) tags to map the eQTL responsible for inter-generic differences in cichlid opsin expression. Our results identify numerous overlapping eQTL which suggest that divergence in opsin *trans*-regulatory loci may be as important to the evolution of color vision as structural mutations within the opsins themselves.

## Results and discussion

### Genetic cross and opsin gene expression

**Figure 1:**
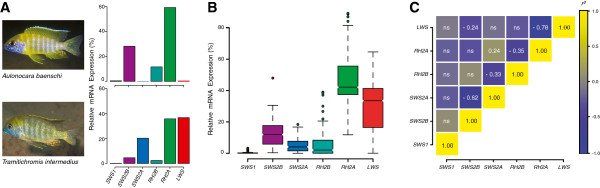
**Interspecific divergence in opsin gene expression among African cichlids and their hybrid progeny. [A]***A. baenschi* and *T. intermedius* differ qualitatively in their patterns of opsin gene expression^a^. **[B]** Box-and-whisker plots of opsin expression in the hybrid F_2_ progeny (n=115). The notched line defines the location of the median; the boxes define the upper and lower interquartile range (IQR); and the whiskers define observations within the 1.5 IQR. **[C]** Heatmap of correlation coefficients for F_2_ opsin expression show that the expression of the *SWS2B/SWS2A* and *RH2A/LWS* opsins are highly correlated. Numbers indicate *r*^*2*^ values for pairwise comparisons with P < 0.05. ^a^ Images of *A. baenschi* and *T. intermeidus* by Ad Konings, used with permission.

We sampled 115 F_2_ progeny from an experimental cross of two Lake Malawi cichlids that differ in opsin gene expression. *Aulonocara baenschi* express the middle wavelength-sensitive opsin palette (*SWS2B*, *RH2B*, and *RH2A*) while *Tramitichromis intermedius* express the long wavelength palette (*SWS2A*, *RH2A*, and *LWS*) [17] (Figure 1A). Using quantitative PCR, we measured the expression of all seven opsins (combining *RH2A-α* and -*β*, which have similar absorbance spectra [15]) and found that they varied continuously among the F_2_, except for *SWS1*, which was not expressed by either *A. baenschi* or *T. intermedius* (Figure 1B) [13]. As expected from our previous evolutionary and developmental studies [7,13,16,19,20], we found that the expression of the *SWS2B/SWS2A* and *RH2A/LWS* opsin pairs are each negatively correlated in a compensatory manner (Figure 1C). Although some degree of autocorrelation between opsins is expected since we measure the expression of each gene relative to the total expression of all six opsins, compensatory trade-offs between these opsin pairs are also expected since they must be alternatively expressed within identical photoreceptor classes (single- and double-cones, respectively) in order to form the distinct visual palettes found among different cichlid species. The evolutionary and developmental correlation of these pairs of opsins suggests that their expression is likely controlled by physically linked or pleiotropic loci.

### Genotyping and validation of SNPs via RAD-seq

Restriction-site associated DNA sequencing (RAD-seq) is a relatively new and cost-effective method of simultaneously identifying and genotyping hundreds of single nucleotide polymorphisms (SNPs) in non-model species [21]. For our cichlid cross, we generated reduced-representation RAD-seq libraries for all 115 individuals and sequenced these for 100 cycles on the Illumina HiSeq2000 platform. Sequencing yielded an average of 3.14 million (± 0.17 million SEM) reads per individual among ~ 66,500 unique *SbfI* restriction sites (see Additional file 1). We used the program Stacks v0.996 [22] to match orthologous RAD-tags between the P_0_, identify alternatively-fixed SNPs, and infer genotypes at these sites among the F_2_. We filtered these SNPs for genotyping completeness, adherence to Hardy-Weinburg equilibrium, coverage, and unambiguous placement within a draft assembly of the *Metriaclima zebra* cichlid genome (assembly available at the Cichlid Comparative Genome Browser, Bouillabase.org). We ultimately retained a conservative set of 562 SNPs for linkage mapping and QTL analysis. The average coverage of each genotyped SNP was 45x (± 2x, SEM) in the F_2_ and 130x in the P_0_, while the average genotype completeness was 66%. In addition to the RAD-seq loci, we also genotyped microsatellite and SNP markers for ten *trans-*regulatory factors that have been shown to influence vertebrate opsin expression [10], including members of the thyroid hormone and retinoic acid family of transcription factors, retinoic acid related orphan receptors, photoreceptor-specific nuclear receptors, and steroid co-activators. We also included one microsatellite marker for each tandem array of the opsin genes located on cichlid linkage group (LG) 5 (Additional file 2).

Because RAD-seq is a relatively new method, there are currently no published estimates of this method’s accuracy. To estimate the potential error-rate of our study, we validated the genotypes inferred from eleven RAD-seq markers via PCR and direct cycle sequencing. We found errors in the genotypes inferred from all eleven loci, regardless of whether or not the marker passed our conservative filtering criteria (Additional file 3). We estimate that the average genotyping error rate of these eleven markers is 11.07±1.36% (min = 3.23%, max = 20.59%). In particular, the genotypes inferred from RAD-seq underestimated the true number of heterozygotes found by cycle sequencing, suggesting that reads containing alternate alleles were either (a) disproportionally amplified and sequenced, or (b) misassembled into separate RAD-seq loci. The accuracy of RAD-seq can be influenced by numerous technical and user-related factors, and an analysis of the precise source of this genotyping error is out of the scope of our present study. However, we do present an expanded discussion of this problem in Additional file 4. Although this is the first published estimate of the accuracy of RAD-seq, a similar analysis of a second cross of cichlids from Lake Malawi presented data which suggests that 3.13% of genotypes for the P_0_ were incorrectly inferred to be homozygous [23]. Although disconcerting, we present evidence that these errors do not significantly hinder our ability to detect eQTL for opsin expression (Additional file 3; see below).

### Both *cis-* and *trans-* regulatory eQTL contribute to divergence in cichlid opsin expression

We assembled the RAD-seq and candidate gene loci into a high-density linkage map of the cichlid genome, and then we refined the placement of markers in this map by aligning the 100 bp consensus sequence of each RAD-seq tag to draft assemblies of the *Oreochromis niloticus* and *Metriaclima zebra* cichlid genomes (Bouillabase.org). The resulting genetic map comprised 23 linkage groups spanning 1669 cM (Additional file 5; Additional file 6). This map is largely co-linear with a genetic map of the model cichlid *O. niloticus*, which has a haploid genome of 22 chromosomes and a combined length of 1.1 Gb and 1331 cM [24]. All linkage groups in our map share the same numbering scheme as those for *O. niloticus*, with the sole exception of the twenty-third linkage group, LG UN.

**Figure 2:**
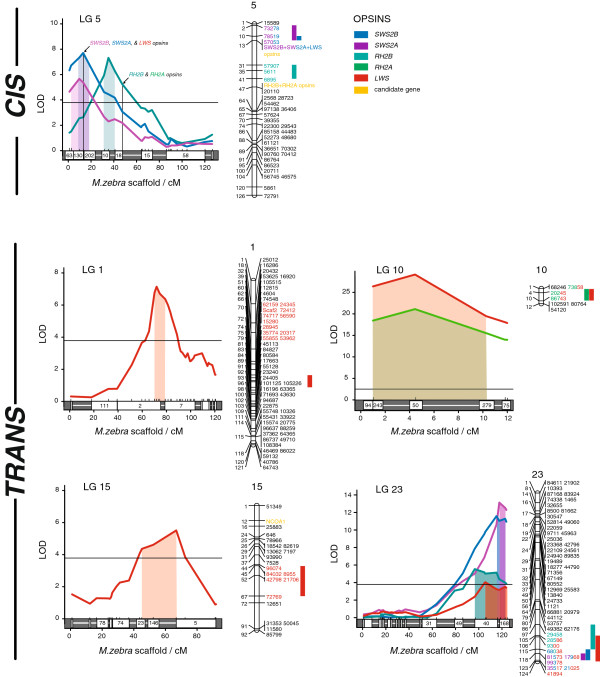
**Prominent role for *trans*-regulatory divergence in the evolution of cichlid opsin expression. **Divergence at 11 overlapping eQTL located both *cis-* (LG 5) and *trans*- (LGs 1, 10, 15, and 23) to the opsins contributes to interspecific differences in cichlid opsin expression and color vision (n = 115). eQTL on linkage group 5 are considered potential *cis*-regulatory loci given their linkage to the opsin genes on this chromosome; however, it is also possible that some or all of these regions contain diffusible regulatory elements that act in *trans*. LOD plots of eQTL and their associated opsins are shown for significant eQTL only. Horizontal bars represent the 95^th^ percentile of genome-wide LOD scores (LOD = 3.7) and colored rectangles delineate the 95% Bayesian confidence intervals for each eQTL. Tic-marks above the x-axis indicate the position of each genetic marker, while the white boxes indicate the genomic scaffolds that these markers map to within the *Metriaclima zebra* cichlid genome assembly (not to scale). A linkage map with marker names is shown to the right of each LOD plot. Colored bars and highlighted marker names correspond to the 95% Bayesian confidence intervals shown in the LOD plots (for LG 10, drop 2-LOD are used).

Previously, we estimated that 6–12 eQTL underlie inter-generic differences in opsin gene expression between *A. baenschi* and *T. intermedius*[17]. Specifically, we estimated that 1–2 eQTL each underlie the divergent expression of the *SWS2A*, *SWS2B*, *RH2B*, and *RH2A* opsins, and that 4–5 eQTL underlie the divergent expression of the *LWS* opsin. In this study, we used a model-selection approach to multiple QTL mapping implemented in the program R/qtl in order to identify as many eQTL as possible, including their interactions, while minimizing the inclusion of false-positives [25,26]. This approach identified a total of 11 eQTL: 2 each for *SWS2B*, *SWS2A*, and *RH2B* expression, 1 for *RH2A* expression, and 4 for *LWS* expression, closely matching our previous estimate (Figure 2, Table 1). These models explained a total of 27.77%, 48.74%, 11.25%, 57.05%, and 78.05% of the variation in the expression of the *SWS2B*, *SWS2A*, *RH2B*, *RH2A*, and *LWS* opsins, respectively. None of the models included epistatic (interactive) effects, although several eQTL showed considerable deviations from additivity (Table 1). On average, each eQTL explained 21.57% (±5.23 SEM) of the variance in the expression of the affected opsin and had a logarithm of the odds (LOD) score equal to 10.75±2.35. The majority of the eQTL we found overlap each other on just two cichlid linkage groups, LGs 5 and 23, although we also found eQTL on LGs 1, 10, and 15 (Figure 2).

**Table 1:** **Summary of *cis*- and *trans-*regulatory eQTL detected for cichlid opsin expression**^**b**^

**Linkage**	***cis *or**	**Opsin**	**RAD**	**Position**	**LOD**	**PVE**	**Dom.**	**Add.**	**Dom.**	**95% BCI**	**95% BCI**
**group**	***trans***^**b**^	**gene**	**tag**	**(cM)**	**score**	**(%)**	**dev.**	**effect**	**effect**	**(cM)**	**(markers)**
1	*trans*	LWS	56590	71.87	7.14	7.27	2.44	−6.96	0.96	70.16-79.00	62159-53962
5	*cis*	SWS2B	78519	9.65	5.66	10.87	1.73	−3.99	1.87	2.48-13.09	73728-RBusat
5	*cis*	SWS2A	57053	13.09	7.70	18.52	0.39	2.82	0.75	9.65-13.09	73728-RBusat
5	*cis*	RH2B	5611	35.30	7.32	21.70	−3.05	−6.27	−2.70	31.41-40.84	57907-6895
10	*trans*	RH2A	20245	4.44	21.1	57.05	−9.44	−17.52	−10.69	1.00-10.27	73858-86743
10	*trans*	LWS	20245	4.44	29.14	48.55	11.26	16.26	11.70	1.00-10.27	73858-86743
15	*trans*	LWS	72769	66.84	5.51	5.42	−11.60	−0.09	−9.86	45.13-66.85	84032-72769
23	*trans*	SWS2B	99378	117.70	13.13	17.81	0.03	−5.34	0.04	117.69-122.74	81573-35517
23	*trans*	SWS2A	68038	115.29	11.58	30.24	−1.74	3.61	−0.52	115.28-122.74	68038-21025
23	*trans*	RH2B	29458	96.56	5.54	15.82	−3.32	−4.28	−5.13	96.55-115.29	29458-68038
23	*trans*	LWS	9300	105.60	4.41	4.04	6.06	2.69	6.06	104.84-123.80	28586-41894
Avg.	-	-	-	-	10.75	21.57	[4.64]	[6.35]	[4.57]	11.16	-

^b^ We conservatively classify all eQTL that occur on the same linkage group as the opsins as *cis*, while the eQTL found on unlinked chromosomes are necessarily *trans*.

The five opsin genes that vary in expression between *A. baenschi* and *T. intermedius* are all found in two tandem arrays on LG 5 (Figure 2); thus, the three eQTL on this LG represent potential *cis-*regulatory eQTL given their proximity to the opsins (Figure 2). Two eQTL for *SWS2B* and *SWS2A* expression on LG 5 each include a microsatellite marker located < 1 cM from the *SWS2BSWS2ALWS* opsin gene array. A third eQTL for *RH2B* expression is also located on LG 5, but it does not overlap with the other two eQTL and it does not include the microsatellite marker for the *RH2B-RH2Aα-RH2Aβ* array (Figure 2). Although we consider these three eQTL putative *cis*-regulatory loci, we note that *trans-*regulatory factors such as microRNAs also occur in close physical proximity to the cichlid opsin genes [18]. Additionally, the peak of the *RH2B* eQTL is located far (12 cM or ~ 9 Mb) from the *RH2B* opsin. Therefore, it is possible that one or more of the eQTL on LG 5 actually represent divergent *trans*-regulatory factors. The presence of these three eQTL partially confirms the preliminary mapping results of Carleton et al. [17] who also found evidence of eQTL for these three opsins on LG 5, although we did not find comparable evidence for an *SWS2B/SWS2A* eQTL on LG 13 as in [17].

The remaining eQTL on LGs 1, 10, 15, and 23 represent divergent *trans*-regulatory loci since no opsin genes are located on these four linkage groups (the remaining opsin, *SWS1*, is located on LG 17 [17]). Two of the eQTL for *LWS* opsin expression are found alone on LGs 1 and 15, and a third was found on LG 10, where it overlaps perfectly with the sole eQTL for *RH2A* expression (Figure 2; Table 1). The fourth and final eQTL for *LWS* expression was found on the distal arm of LG 23, where it overlaps three other eQTL for *SWS2B*, *SWS2A*, and *RH2B* expression (Figure 2).

To confirm the results of our eQTL mapping analysis, we re-sequenced the RAD-seq loci underlying six eQTL and used single-marker regression to confirm these associations (Additional file 3). Our cycle sequencing results confirm the presence of eQTL for *RH2B*, *RH2A/LWS*, and *SWS2B/SWS2A/RH2A* expression on LGs 5, 10, and 23, respectively (ANOVA, all *F* > 8.45, all *P* < 0.006; see Additional file 3), while three additional eQTL for *LWS* and *SWS2B/SWS2A* expression on LGs 1 and 5 are also confirmed by candidate microsatellite loci (Figure 2). Since we found numerous eQTL that match our expectations from previous Castle-Wright estimates [17] and support these associations with re-sequencing, it seems unlikely that the genotyping errors inferred from RAD-seq impaired out ability to detect QTL in this study.

### A genetic regulatory network for cichlid opsin expression

Although experimental work in humans, mammals, and fish has identified many *cis*- and *trans*-regulatory elements that control opsin gene expression and photoreceptor identity (e.g., [8-10]), our study has identified those elements of the regulatory network that allow for rapid evolutionary changes in cichlid opsin gene expression and color vision, many of which have occurred in only the last two million years. In particular, the overlapping eQTL on LGs 5, 10, and 23 suggests that many opsins may be co-regulated by tightly linked or pleiotropic loci. Consistent with the hypothesis of compensatory regulation, we find that the overlapping eQTL for *SWS2B/SWS2A* and *RH2A*/*LWS* expression are predominantly additive, with effects that are similar in magnitude but opposite in direction (Table 1; Additional file 7). The distinct “visual palettes” of African cichlids could therefore be explained by the presence of transcription factors or regulatory “hot spots” that control the compensatory expression of two or more opsins simultaneously. Unfortunately, however, none of the 10 candidate genes used in our analysis were found within these eQTL regions (Figure 1). But by mapping the RAD-seq markers from these regions to the *M. zebra* genome assembly, we found that the 95% Bayesian confidence intervals for most eQTL correspond to just one or two genomic scaffolds (Figure 2). A quick survey of genes located within these regions reveals additional candidates for photoreceptor function and opsin gene expression, including *GNAO1*, *NEUROD1*, and *PAX6* (LG 1), *NCOA1* and *RAR-γ2* (LG 5), *PRPH2* and *OTX-2* (LG 10), *NR2E1* (LG 15), and *RXR-α* (LG 23) [10]*.* Future work will fine-map the causative mutations within these eQTL and use additional crosses to identify loci responsible for regulatory variation in the remaining *SWS1* opsin gene. These future mapping studies should reveal more about the architecture and evolution of the genetic regulatory network governing vertebrate opsin gene expression.

### Comparison of opsin structural and regulatory mutations

Recent reviews of genetic adaptation have contrasted the role that different structural and regulatory mutations play in the evolution of morphological and physiological diversity [27-30]. To be sure, structural mutations within the opsins certainly contribute to interspecific differences in vertebrate photoreceptor sensitivity and color vision [1,3,4,14]. In cichlids, *A. baenschi* and *T. intermedius* exhibit amino acid substitutions within several opsins (see Additional file 3 within [13]), including one, *SWS2B* A269T, that has been shown to shift the sensitivity of this opsin by 10 nm in some fish species [31]. But regulatory mutations can also tune color vision by altering the ratio of photoreceptors that contain paralogous opsins of different sensitivities [5-7]. Compared to the presumably functional A269T mutation within the *SWS2B* opsin, we find that the overlapping regulatory eQTL for *SWS2B/SWS2A* expression on LGs 5 and 23 shift the expression of these opsins by an average of 6.81% and 8.95% in homozygotes, respectively (see the additive effect of each locus in Table 1 and the effect plots in Additional file 7). Although small, these regulatory changes are expected to shift the average absorbance of *SWS2*-containing photoreceptors by 9.00 nm and 11.90 nm – a shift that is equal to that caused by the *SWS2B* A269T structural mutation. This shift will be even greater if considered in combination with the other overlapping eQTL for *RH2B* and *LWS* expression on LG 23 (Figure 2). Thus, the evolution of color vision could result from regulatory as well as structural mutations, especially among species that possess several paralogous opsin genes.

### Comparison of opsin *cis-* and *trans*-regulatory mutations

In addition to comparisons of structural and regulatory mutation, reviews of regulatory evolution have also contrasted the likely impact of *cis*- and *trans*-regulatory mutations on expression phenotypes [27,30,32]. These studies have emphasized a key role for *cis*-regulatory evolution and the past decade of research has uncovered a growing number of *cis*-regulatory mutations responsible for morphological adaptations among divergent but closely related species (e.g., [33-36]). One reason for this emphasis is undoubtedly discovery bias. By definition, *cis*-regulatory mutations occur in close proximity to the genes they regulate; therefore, they are better candidates for genetic mapping than *trans*-regulatory mutations, which could be located anywhere in the genome. But a second, more compelling reason for this emphasis is the unique action of *cis*-regulatory alleles. *Cis*-regulatory alleles are typically additive and modular in their effect. These two features increase the chance that alternate *cis-*regulatory alleles can reach fixation via natural selection, and they also minimize any negative consequences of pleiotropy that could result from structural mutations within the developmental regulatory genes that typically govern morphological evolution [27,30]. Furthermore, although the potential for mutation is probably greater for *trans-* rather than *cis-*regulatory sequences [37], direct comparisons within and between species suggest that *trans*-regulatory mutations are initially more common for intraspecific comparisons, but that *cis*-regulatory mutations accumulate preferentially over time and dominate interspecific and intergeneric comparisons [38-43]*.* Thus, both theory and observation suggest that *cis*-regulatory mutations should preferentially contribute to interspecific divergence in gene expression and phenotypic adaptation, and examples of this have become increasingly common, while examples of phenotypic adaptation due to *trans*-regulatory mutations are exceptionally rare (e.g., [44,45]).

However, here we study inter-generic divergence in cichlid opsin expression and find that *trans*-regulatory loci account for the majority (8/11 or 73%) of the eQTL identified. Additionally, these *trans*-regulatory eQTL contribute to the divergent expression of all five variable opsins (Figure 2, Table 1), whereas the potential *cis*-regulatory eQTL on LG 5 only contribute to the divergent expression of three opsins, and then only in combination with other *trans*-regulatory eQTL (Figure 2). Thus, our results suggest that *trans*-regulatory variation in gene expression may contribute to phenotypic evolution more commonly than expected, and that the significance of *trans*-regulatory divergence need not be limited to intraspecific variation. In the future, allele-specific expression can be used to better proportion interspecific variation in cichlid opsin expression among different *cis*- and *trans*-regulatory factors; however, our current eQTL analysis indicates that divergence in *trans-*regulatory loci disproportionately contribute to the evolution of opsin gene expression and photoreceptor sensitivity among African cichlid fishes.

Five factors likely underlie our finding that *trans*-regulatory eQTL disproportionately contribute to the evolution of cichlid color vision; two factors are related to our study design and three to real biological phenomena. First, by measuring opsin gene expression directly, we can easily proportion vision QTL into potential *cis*- and *trans*-regulatory loci based on their genomic position relative to the opsin genes. Studies that examine the sequence and expression of genes related to specific phenotypes, like the opsins and color vision, may be better suited to tease apart the role of different structural and regulatory mutations than studies that simply start with morphological or physiological traits and map those easiest to identify (for an example, see [37]). Second, the cichlids of Lake Malawi are the product of an adaptive radiation that has produced hundreds of new species in less than 1 million years [46]. Many cichlid species and genera remain interfertile and share numerous polymorphisms, possibly making interspecific comparisons of regulatory divergence among cichlids more analogous to intraspecific comparisons among other model species [47]. Third, the opsin genes have a limited physiological function. They encode structural proteins that are not regulatory, and their expression is generally restricted to the neural retina and skin [48], although opsins are also expressed within the brain and ovaries of some fish species [49]. This limited function means that either structural, *cis*-, or *trans*-regulatory mutations could be used to tune photoreceptor sensitivity while still avoiding the negative consequences of pleiotropy that are supposed to favor *cis*-regulatory alleles [27,29,30]*.* Fourth, the opsin genes occur at the end of a large regulatory network that includes multiple interacting transcription factors, including the retinoic acid and thyroid hormone nuclear receptors [10]. Since the number of transcription factors that control a gene’s expression is positively correlated with the degree of *trans*-regulatory divergence [39], the large number of *trans*-regulatory factors that control vertebrate opsin expression suggests that these genes could be predisposed to evolve new expression patterns in *trans*. Finally, the distinct “visual palettes” of African cichlids are the result of heterochronic changes that simultaneously alter the expression of multiple opsins genes. Phenotypes intermediate to these three palettes are generally not found among wild-caught species [13,16]. Theoretically, the coordinated compensatory changes required to produce these palettes could be easily achieved by a mutation that alters the structure or expression of a single *trans*-regulatory factor that controls multiple downstream opsins. In contrast, separate mutations upstream of each opsin would be required to make the same changes via *cis*-regulatory divergence, especially for opsins that are not joined in a tandem array. Thus, *trans*-regulatory mutations may be better suited to the evolution of complex phenotypes that are governed by the coordinated expression of multiple genes, like those that govern many sensory systems.

## Conclusions

Although the evolution of color vision in vertebrates is primarily attributed to structural mutations within the opsin genes, we have shown that divergence in both *cis*- and *trans*-regulatory loci can also tune vertebrate photoreceptor sensitivity. In particular, we found that divergence in *trans-*regulatory eQTL disproportionally contribute to the inter-generic evolution of opsin gene expression and photoreceptor sensitivity among African cichlid fishes. Although several unique properties of the opsins may explain this observation, our results suggest that *trans*-regulatory mutations could contribute to phenotypic divergence more commonly than previously expected, especially in cases where the coordinated and compensatory expression of multiple genes are necessary to drive the evolution of phenotypic traits.

## Methods

### Cross design and phenotyping

We sampled 115 progeny from three *A. baenschi* x *T.intermedius* F_2_ crosses previously described in Carleton et al. [17]. Each cross had the same *T. intermedius* grandfather but unique *A. baenschi* grandmothers. We used 72, 23, and 20 F_2_ from each cross, respectively. We raised all progeny to sexual maturity (>6 months post fertilization) and then euthanized them at approximately the same time of day using buffered MS-222 following University of Maryland IACUC protocol R-09-73. Once euthanized, we clipped fin tissue for DNA and dissected retinas from each fish. We then extracted total RNA from the retinas, which we reverse-transcribed to cDNA and used to measure the expression of the opsin genes via quantitative real-time PCR (grouping the genetically and functionally similar *RH2A-α* and -*β* opsins). These methods followed our previously published protocols [13,15,17].

### RAD-seq library construction and genotyping

Our methods for RAD-seq library construction followed the protocols of Etter et al. [21]. We extracted genomic DNA from 10–20 mg fin tissue from each fish using a DNeasy Blood & Tissue extraction kit and 4.0 μL RNAse (Qiagen). We estimated the concentration of each DNA sample using the Quant-iT™ dsDNA High-Sensitivity Kit (Invitrogen) and a BioTek FLx800 Fluorometer. Next, we digested 1 μg of DNA from each individual in a 50 μL restriction reaction of 10 units (U) *Sbf*I-HF (New England Biolabs [NEB]) and NEBuffer4 (NEB) following the manufacturers protocols. We then ligated a modified Solexa^©^ adaptor to each sample (P1, 2006 Illumina, Inc., all rights reserved; top: 5^′^-AATGATACGGCGACCACCGAGATCTACACTCTTTCCCTACACGACGCTCTTCCGATCTxxxxxTGCA-3^′^; bottom: 3^′^-TTACTATGCCGCTGGTGGCTCTAGATGTGAGAAAGGGATGTGCTGCGAGAAGGCTAGAxxxxx-5^′^), where xxxxx denotes one of 32 5-bp barcodes used to identify the sequences of each individual within a library (Additional file 8). We then combined 3.75 μL (0.0625 μg) DNA from the reactions of 32 individuals into a common 120 μL pooled library and randomly sheared them to an average length of 500 bp using a UCD-300 Biorupter (Diagenode) on high for 5 cycles of 30 seconds ON and 30 seconds OFF. We ran the contents of each library on a separate 1.25% agarose gel and excised DNA fragments between 300 bp and 650 bp and cleaned them using a MinElute Gel Extraction kit (Qiagen). Next, we repaired the blunt ends of each DNA library with a 10X Quick Blunting enzyme kit (NEB), cleaned the reaction with a QiaQuick PCR Purification kit, then added adenine overhangs to the 3^′^ end of the DNA fragments using Klenow 3^′^- 5^′^ exo (NEB). We once again cleaned the reaction with a QiaQuick PCR Purification kit, then we ligated a second modified Solexa© adaptor (P2, 2006 Illumina, Inc., all rights reserved; top: 5^′^-Phos-GATCGGAAGAGCGGTTCAGCAGGAATGCCGAGACCGATCAGAACAA-3^′^; bottom: 3^′^-TCTAGCCTTCTCGCCAAGTCGTCCTTACGGCTCTGGCTAGAGCATACGGCAGAAGACGAAC-5′) to the DNA. Finally, we PCR amplified 2 μL of each raw library in a 50 μL reaction using modified Solexa© amplification primers (2006, Illumina, Inc., all rights reserved; P1 forward: 5^′^-AATGATACGGCGACCACCGA-3^′^; P2 reverse: 5^′^-CAAGCAGAAGACGGCATACGA-3^′^), and 2U Phusion Taq with 5X Phusion HF Buffer. The sole exception between our method and the one described in Etter at al. [21] is that we PCR amplified our reduced representation RAD-seq libraries for 19 cycles instead of 18. We gel-extracted each PCR amplified library from a separate 1% agarose gel, cutting out DNA fragments between 300 bp and 800 bp. We purified each extracted library with a MinElute Gel Extraction kit, then eluted the final libraries with 20 μL Buffer EB. We quantified the concentration of each library with Quant-iT™ and the BioTek fluorometer, then diluted the final library to 10 nM and sent them to the University of Oregon for sequencing.

Following sequencing, we filtered the reads for quality (Q20 across 90% of the 100 bp read) and the presence of a complete *SbfI* site and 5-bp barcode (Additional file 8). We then used the program Stacks v0.996 [22] to assemble the reads and infer genotypes for linkage and QTL mapping. The Stacks script *denovo_map.pl* was used to (a) assemble the reads from the P_0_ and F_2_ into unique loci and identify potential heterozygous alleles, (b) aggregate the stacks from the P_0_ of each family into a separate catalog of orthologous loci and identify SNPs between them, and (c) match these loci back to their respective progeny and infer genotypes at loci containing SNPs. Because we generally expect to find only one polymorphism every 1 Kb between cichlid species [47], we allowed for one mismatch when assembling the 100 bp reads into loci and when matching orthologous loci among the P_0_ and F_2_ progeny. Additionally, we also chose conservative parameters to infer genotypes from the RAD-seq loci. We modified the default parameters of the Stacks script *genotypes* to set *min_hom_seqs* = 20, *max_het_seqs* = 0.08 or 2/25, and *min_het_seqs* = 0.02 or 1/50, where *min_hom_seqs* is the minimum number of sequencing reads required to call a genotype homozygous in the F_2_, *max_het_seqs* is the minimum ratio of the depth of the minor allele to the major allele required to call a genotype heterozygous, and *min_het_seqs* is the maximum ratio of the depth of the minor allele to the major allele required to call a genotype homozygous. For the reads that comprise a locus in any given F_2_, if the ratio of the depth of the minor allele to the major allele is greater than *max_het_seqs* (0.08), it is called a heterozygote; if the ratio of the depth of the minor allele to the major allele is less than *min_het_seqs* (0.02), it is called a homozygote; and if the ratio falls between *max_het_seqs* and *min_het_seqs*, it is considered ambiguous and is called as unknown or missing. Following genotyping, we further filtered the markers by excluding those that were (a) not differentially fixed between the parents of at least one cross, (b) genotyped in less than 50% of the F_2_, (c) did not meet the cut-off for Hardy-Weinberg equilibrium following a chi-square test at *P* > 0.0495, and (d) could not be unambiguously mapped to the cichlid genome within a Burroughs Wheeler aligner (BWA) edit distance of ≤2. To fulfill this latter criterion, we mapped the 100 bp consensus sequences from each cross’s *denovo_map* catalog to a draft assembly of the *Metriaclima zebra* genome (M. zebra v0, see the comparative cichlid genome browser at Bouillabase.org) using the program BWA v0.6.1 with default parameters [50]. Finally, since Stacks v0.996 cannot automatically filter the haplotypes of the P_0_ based on the same genotyping parameters specified in *genotypes*, we manually inspected all markers passing the above criteria in the P_0_. We then further filtered markers that did not meet the same *min_hom_seqs*, *max_het_seqs*, and *min_het_seqs* criteria in the P_0_, although we raised the cutoff of *min_het_seqs* to 0.04 or 2/50, since the coverage for each stack was generally much greater in these individuals. The composition of genotypes in our final data set was 28.5% AA, 42.7% AT, and 28.8% TT.

### Candidate genes markers and RAD re-sequencing

We identified the location of several candidate genes for vertebrate opsin regulation in the *Gasterosteus aculeatus* genome (Broad/gasAcu1 assembly, Feb. 2006), then used the resulting coordinates to search for sequences from a genome assembly of the cichlid *Oreochromis niloticus* aligned to *G. aculeatus* ([51]; see the comparative cichlid genome browser at Bouillabase.org). We used the programs Perfect Microsatellite Repeat Finder (http://sgdp.iop.kcl.ac.uk/nikammar/repeatfinder.html) and Sequencher® to identify microsatellite and SNP sequences within 600 kb (< 1 cM) of each gene, then designed primers and fluorescently-labeled probes for PCR with the program Primer3Plus [52]. We used the same method to generate PCR and sequencing primers for 11 RAD-seq loci. Following PCR, we cycle sequenced candidate and RAD-seq loci containing SNPs using the BigDye terminator v3.1 cycle sequencing kit (Applied Biosystems, ABI). We ran the products of all microsatellite and SNP reactions on an ABI 3730xl Genetic Analyzer and genotyped them using GeneMapper® (ABI) and Sequencher® (GeneCodes) software. Additional file 2 lists all candidate gene and re-sequencing primers used.

### Linkage mapping and eQTL analysis

We used the program R/qtl [25] to assemble the RAD-seq and candidate loci into a genetic linkage map of the cichlid genome and scan for QTL associated with opsin gene expression. We assembled markers into linkage groups (LG) based on recombination fractions after specifying a minimum LOD cutoff of 4.35, which is the cutoff that yielded the ~ 22 LG expected from the genome of the model cichlid *Oreochromis niloticus*[24]. We then determined the order of markers along each LG by rippling their position within a window of seven markers and chosing the order that required the smallest number of obligate crossovers. We further refined this order by manually grouping markers that mapped to the same scaffold of the *Metriaclima zebra* genome assembly and then estimated the final inter-marker distances using the Carter-Falconer map function [53]. We used the comparative cichlid genome browser to BLAST the consensus sequence of each marker to the anchored assembly of the *Oreochromis niloticus* cichlid genome ([54]; assemblies available at Bouillabase.org) in order to infer the orthology of each LG relative to the genetic map of *O. niloticus*[24].

After linkage mapping, we scanned the cichlid genome for eQTL associated with opsin expression using *stepwiseqtl*, a model-selection approach to multiple-interval-mapping implemented in R/qtl. This method is described in detail by Broman and Sen [25]. For this analysis, we specified the use of Haley-Knott regression when calculating the LOD of eQTL. We restricted all eQTL positions to genotyped markers only – we did not impute inter-marker loci at fixed distances in the genome – but we did fill in missing genotypes at marker positions using simulations based on the genotypes of near-by markers. Finally, we also used 1000 permutations of the data among hybrid families in order to account for family structure when estimating penalties for incorporating additional eQTL into the forward and backward selection models and when estimating the 95^th^ percentile of genome-wide LOD scores under a null model of no eQTL.

### SWS2-photoreceptor sensitivity

Finally, we estimated the impact of changes in opsin gene expression on the average sensitivity of *SWS2*-containing photoreceptors by calculating the average wavelength of maximum absorbance of these photoreceptors weighted by the maximum absorbance of the *SWS2B* and *SWS2A* opsins and their relative level of expression in *T. intermedius* homozygotes. We describe this method in several previous publications [7,13,16,20]. In general, this method produces results that are similar to values of photoreceptor absorbance determined physiologically [7].

## Abbreviations

eQTL: Expression Quantitative Trait Loci; LOD: Logarithm of the Odds; SWS: Short wavelength sensitive opsin; RH2: Rhodopsin-like opsin; LWS: Long wavelength sensitive opsin; RAD: Restriction site-associated DNA; SEM: Standard error of the mean; BCI: Bayesian credible interval.

## Competing interests

The authors declare that they have no competing interests.

## Authors’ contributions

KEO helped design and perform the research, analyze data, and write the paper; JES, ZP, NK, ZN, and HW helped perform the research; MAC helped analyze the data; and KLC helped design and perform research, analyze data, and write the paper. All authors read and approved the final manuscript.

## Supplementary Material

Additional file 1 **Excel file of sequencing and assembly statistics of all P**_**0 **_**and F**_**2 **_**used in this study.** Click here for file

Additional file 2 Table of primers used to amplify and genotype polymorphisms at candidate gene markers and re-sequence RAD-seq loci. Click here for file

Additional file 3 **Table of genotyping errors for 11 markers inferred from RAD-seq, including updated association values^c^. **^c^ P-value following ANOVA of cycle-sequencing genotypes and opsin expression of 5611 with *RH2B*; 20245 with *RH2A* and *LWS*; and 28586 with *SWS2B*, *SWS2A* and *RH2B*. Click here for file

Additional file 4 Supplemental text discussing genotyping error and RAD-seq. Click here for file

Additional file 5 Linkage map of the cichlid genome along with eQTL positions for cichlid opsin gene expression. Click here for file

Additional file 6 Table of the identity, sequence, and map-position of all RAD-seq markers used in this study. Click here for file

Additional file 7 **Distribution of opsin gene expression values among genotypic classes at eQTL loci. **Genotypic classes include individuals that are homozygous for *Aulonocara baenschi* alleles (AA), homozygous for *Tramitichromis intermedius* alleles (TT), or heterozygous (AT). Marker loci are from the eQTL peaks indicated in Figure 2 and Table 2. Bars represent mean ± 1 SEM. The number of individuals varies among plots, though the average number of individuals is n = 62.60±4.06 SEM. Click here for file

Additional file 8 **Excel file of 32 5-bp barcodes used to label F**_**2 **_**DNA in each reduced-representation RAD-seq library.** Click here for file

## References

[B1] Yokoyama S Evolution of dim-light and color vision pigments Annu Rev Genomics Hum Genet 2008 9 259 282 10.1146/annurev.genom.9.081307.164228 18544031

[B2] Wald G The molecular basis of visual excitation Nature 1968 219 5156 800 807 10.1038/219800a0 4876934

[B3] Neitz M Neitz J Jacobs GH Spectral tuning of pigments underlying red-green color vision Science 1991 252 5008 971 974 10.1126/science.1903559 1903559

[B4] Yokoyama S Zhang H Radlwimmer FB Blow NS Adaptive evolution of color vision of the Comoran coelacanth (Latimeria chalumnae) Proc Natl Acad Sci USA 1999 96 11 6279 6284 10.1073/pnas.96.11.6279 10339578 PMC26872

[B5] Fuller RC Carleton KL Fadool JM Spady TC Travis J Genetic and environmental variation in the visual properties of bluefin killifish, Lucania goodei J Evol Biol 2005 18 3 516 523 10.1111/j.1420-9101.2005.00886.x 15842481

[B6] Hagstrom SA Neitz J Neitz M Variations in cone populations for red-green color vision examined by analysis of mRNA Neuroreport 1998 9 9 1963 1967 10.1097/00001756-199806220-00009 9674575

[B7] Parry JW Carleton KL Spady T Carboo A Hunt DM Bowmaker JK Mix and match color vision: tuning spectral sensitivity by differential opsin gene expression in Lake Malawi cichlids Curr Biol 2005 15 19 1734 1739 10.1016/j.cub.2005.08.010 16213819

[B8] Hsiau TH Diaconu C Myers CA Lee J Cepko CL Corbo JC The cis-regulatory logic of the mammalian photoreceptor transcriptional network PLoS One 2007 2 7 e643 10.1371/journal.pone.0000643 17653270 PMC1916400

[B9] Nathans J Davenport CM Maumenee IH Lewis RA Hejtmancik JF Litt M Lovrien E Weleber R Bachynski B Zwas F Molecular genetics of human blue cone monochromacy Science 1989 245 4920 831 838 10.1126/science.2788922 2788922

[B10] Swaroop A Kim D Forrest D Transcriptional regulation of photoreceptor development and homeostasis in the mammalian retina Nat Rev Neurosci 2010 11 8 563 576 10.1038/nrn2880 20648062 PMC11346175

[B11] Kocher TD Adaptive evolution and explosive speciation: the cichlid fish model Nat Rev Genet 2004 5 4 288 298 10.1038/nrg1316 15131652

[B12] Carleton K Cichlid fish visual systems: mechanisms of spectral tuning Integr Zool 2009 4 1 75 86 10.1111/j.1749-4877.2008.00137.x 21392278

[B13] Hofmann CM O’Quin KE Marshall NJ Cronin TW Seehausen O Carleton KL The eyes have it: regulatory and structural changes both underlie cichlid visual pigment diversity PLoS Biol 2009 7 12 e1000266 10.1371/journal.pbio.1000266 20027211 PMC2790343

[B14] Terai Y Mayer WE Klein J Tichy H Okada N The effect of selection on a long wavelength-sensitive (LWS) opsin gene of Lake Victoria cichlid fishes Proc Natl Acad Sci USA 2002 99 24 15501 15506 10.1073/pnas.232561099 12438648 PMC137746

[B15] Spady TC Parry JW Robinson PR Hunt DM Bowmaker JK Carleton KL Evolution of the cichlid visual palette through ontogenetic subfunctionalization of the opsin gene arrays Mol Biol Evol 2006 23 8 1538 1547 10.1093/molbev/msl014 16720697

[B16] O’Quin KE Hofmann CM Hofmann HA Carleton KL Parallel evolution of opsin gene expression in African cichlid fishes Mol Biol Evol 2010 27 12 2839 2854 10.1093/molbev/msq171 20601410

[B17] Carleton KL Hofmann CM Klisz C Patel Z Chircus LM Simenauer LH Soodoo N Albertson RC Ser JR Genetic basis of differential opsin gene expression in cichlid fishes J Evol Biol 2010 23 4 840 853 10.1111/j.1420-9101.2010.01954.x 20210829 PMC2996586

[B18] O’Quin KE Smith D Naseer Z Schulte J Engel SD Loh YH Streelman JT Boore JL Carleton KL Divergence in cis-regulatory sequences surrounding the opsin gene arrays of African cichlid fishes BMC Evol Biol 2011 11 120 10.1186/1471-2148-11-120 21554730 PMC3116502

[B19] Carleton KL Parry JW Bowmaker JK Hunt DM Seehausen O Colour vision and speciation in Lake Victoria cichlids of the genus Pundamilia Mol Ecol 2005 14 14 4341 4353 10.1111/j.1365-294X.2005.02735.x 16313597

[B20] Carleton KL Spady TC Streelman JT Kidd MR McFarland WN Loew ER Visual sensitivities tuned by heterochronic shifts in opsin gene expression BMC Biol 2008 6 22 10.1186/1741-7007-6-22 18500997 PMC2430543

[B21] Etter PD Bassham S Hohenlohe PA Johnson E Cresko WA Orgogozo V, Rockman MV SNP discovery and genotyping for evolutionary genetics using RAD sequencing Molecular methods for evolutionary genetics (methods in molecular biology) 2011 New York, NY: Humana Press 519 10.1007/978-1-61779-228-1_9 PMC3658458 22065437

[B22] Catchen JM Amores A Hohenlohe P Cresko W Postlethwait JH Stacks: building and genotyping Loci de novo from short-read sequences G3 (Bethesda) 2011 1 3 171 182 22384329 10.1534/g3.111.000240 PMC3276136

[B23] Parnell NF Hulsey CD Streelman JT The genetic basis of a complex functional system Evolution 2012 Epub ahead of print 10.1111/j.1558-5646.2012.01688.x PMC3490443 23106702

[B24] Lee BY Lee WJ Streelman JT Carleton KL Howe AE Hulata G Slettan A Stern JE Terai Y Kocher TD A second-generation genetic linkage map of tilapia (Oreochromis spp.) Genetics 2005 170 1 237 244 10.1534/genetics.104.035022 15716505 PMC1449707

[B25] Broman KW Sen S A guide to QTL mapping with R/qtl (statistics for biology and health) 2009 New York, NY: Springer

[B26] Manichaikul A Moon JY Sen S Yandell BS Broman KW A model selection approach for the identification of quantitative trait loci in experimental crosses, allowing epistasis Genetics 2009 181 3 1077 1086 10.1534/genetics.108.094565 19104078 PMC2651044

[B27] Carroll SB Evo-devo and an expanding evolutionary synthesis: a genetic theory of morphological evolution Cell 2008 134 1 25 36 10.1016/j.cell.2008.06.030 18614008

[B28] Hoekstra HE Coyne JA The locus of evolution: evo devo and the genetics of adaptation Evolution 2007 61 5 995 1016 10.1111/j.1558-5646.2007.00105.x 17492956

[B29] Stern DL Orgogozo V The loci of evolution: how predictable is genetic evolution? Evolution 2008 62 9 2155 2177 10.1111/j.1558-5646.2008.00450.x 18616572 PMC2613234

[B30] Wray GA The evolutionary significance of cis-regulatory mutations Nat Rev Genet 2007 8 3 206 216 17304246 10.1038/nrg2063

[B31] Cowing JA Poopalasundaram S Wilkie SE Bowmaker JK Hunt DM Spectral tuning and evolution of short wave-sensitive cone pigments in cottoid fish from Lake Baikal Biochemistry 2002 41 19 6019 6025 10.1021/bi025656e 11993996

[B32] Stern DL Orgogozo V Is genetic evolution predictable? Science 2009 323 5915 746 751 10.1126/science.1158997 19197055 PMC3184636

[B33] Chan YF Marks ME Jones FC Villarreal G Jr Shapiro MD Brady SD Southwick AM Absher DM Grimwood J Schmutz J Adaptive evolution of pelvic reduction in sticklebacks by recurrent deletion of a Pitx1 enhancer Science 2010 327 5963 302 305 10.1126/science.1182213 20007865 PMC3109066

[B34] Gompel N Prud’homme B Wittkopp PJ Kassner VA Carroll SB Chance caught on the wing: cis-regulatory evolution and the origin of pigment patterns in Drosophila Nature 2005 433 7025 481 487 10.1038/nature03235 15690032

[B35] Jeong S Rebeiz M Andolfatto P Werner T True J Carroll SB The evolution of gene regulation underlies a morphological difference between two Drosophila sister species Cell 2008 132 5 783 793 10.1016/j.cell.2008.01.014 18329365

[B36] Roberts RB Ser JR Kocher TD Sexual conflict resolved by invasion of a novel sex determiner in Lake Malawi cichlid fishes Science 2009 326 5955 998 1001 10.1126/science.1174705 19797625 PMC3174268

[B37] Gruber JD Vogel K Kalay G Wittkopp PJ Contrasting properties of gene-specific regulatory, coding, and copy number mutations in Saccharomyces cerevisiae: frequency, effects, and dominance PLoS Genet 2012 8 2 e1002497 10.1371/journal.pgen.1002497 22346762 PMC3276545

[B38] Morley M Molony CM Weber TM Devlin JL Ewens KG Spielman RS Cheung VG Genetic analysis of genome-wide variation in human gene expression Nature 2004 430 7001 743 747 10.1038/nature02797 15269782 PMC2966974

[B39] Sung HM Wang TY Wang D Huang YS Wu JP Tsai HK Tzeng J Huang CJ Lee YC Yang P Roles of trans and cis variation in yeast intraspecies evolution of gene expression Mol Biol Evol 2009 26 11 2533 2538 10.1093/molbev/msp171 19648464 PMC2767097

[B40] Tirosh I Reikhav S Levy AA Barkai N A yeast hybrid provides insight into the evolution of gene expression regulation Science 2009 324 5927 659 662 10.1126/science.1169766 19407207

[B41] Wang D Sung HM Wang TY Huang CJ Yang P Chang T Wang YC Tseng DL Wu JP Lee TC Expression evolution in yeast genes of single-input modules is mainly due to changes in trans-acting factors Genome Res 2007 17 8 1161 1169 10.1101/gr.6328907 17615293 PMC1933509

[B42] Wittkopp PJ Haerum BK Clark AG Evolutionary changes in cis and trans gene regulation Nature 2004 430 6995 85 88 10.1038/nature02698 15229602

[B43] Wittkopp PJ Haerum BK Clark AG Regulatory changes underlying expression differences within and between Drosophila species Nat Genet 2008 40 3 346 350 10.1038/ng.77 18278046

[B44] Streisfeld MA Rausher MD Altered trans-regulatory control of gene expression in multiple anthocyanin genes contributes to adaptive flower color evolution in Mimulus aurantiacus Mol Biol Evol 2009 26 2 433 444 10.1093/molbev/msn268 19029190

[B45] Yvert G Brem RB Whittle J Akey JM Foss E Smith EN Mackelprang R Kruglyak L Trans-acting regulatory variation in Saccharomyces cerevisiae and the role of transcription factors Nat Genet 2003 35 1 57 64 12897782 10.1038/ng1222

[B46] Genner MJ Seehausen O Lunt DH Joyce DA Shaw PW Carvalho GR Turner GF Age of cichlids: new dates for ancient lake fish radiations Mol Biol Evol 2007 24 5 1269 1282 10.1093/molbev/msm050 17369195

[B47] Loh YH Katz LS Mims MC Kocher TD Yi SV Streelman JT Comparative analysis reveals signatures of differentiation amid genomic polymorphism in Lake Malawi cichlids Genome Biol 2008 9 7 R113 10.1186/gb-2008-9-7-r113 18616806 PMC2530870

[B48] Ban E Kasai A Sato M Yokozeki A Hisatomi O Oshima N The signaling pathway in photoresponses that may be mediated by visual pigments in erythrophores of Nile tilapia Pigment Cell Res 2005 18 5 360 369 10.1111/j.1600-0749.2005.00267.x 16162176

[B49] Takeuchi Y Bapary MA Igarashi S Imamura S Sawada Y Matsumoto M Hur SP Takemura A Molecular cloning and expression of long-wavelength-sensitive cone opsin in the brain of a tropical damselfish Comp Biochem Physiol A Mol Integr Physiol 2011 160 4 486 492 10.1016/j.cbpa.2011.08.007 21871576

[B50] Li H Durbin R Fast and accurate short read alignment with Burrows-Wheeler transform Bioinformatics 2009 25 14 1754 1760 10.1093/bioinformatics/btp324 19451168 PMC2705234

[B51] Roberts RB Hu Y Albertson RC Kocher TD Craniofacial divergence and ongoing adaptation via the hedgehog pathway Proc Natl Acad Sci USA 2011 108 32 13194 13199 10.1073/pnas.1018456108 21788496 PMC3156204

[B52] Untergasser A Nijveen H Rao X Bisseling T Geurts R Leunissen JA Primer3Plus, an enhanced web interface to Primer3 Nucleic Acids Res 2007 35 Web Server issue W71 74 17485472 10.1093/nar/gkm306 PMC1933133

[B53] Carter T Falconer DC Stocks for detecting linkage in the mouse and the theory of their design J Genet 1951 50 307 323 10.1007/BF02996226 24539711

[B54] Soler L Conte MA Katagiri T Howe AE Lee BY Amemiya C Stuart A Dossat C Poulain J Johnson J Comparative physical maps derived from BAC end sequences of tilapia (Oreochromis niloticus) BMC Genomics 2010 11 636 10.1186/1471-2164-11-636 21080946 PMC3018143

