# Supplementary material for: Evolution of cichlid vision via trans-regulatory divergence
Source: BMC Evol Biol. 2012 Dec 26;12:251. doi: 10.1186/1471-2148-12-251 (PMC3575402; doi:10.1186/1471-2148-12-251)
Supplement: Additional file 3 — Table of genotyping errors for 11 markers inferred from RAD-seq, including updated association valuesc. c P-value following ANOVA of cycle-sequencing genotypes and opsin expression of 5611 with RH2B; 20245 with RH2A and LWS; and 28586 with SWS2B, SWS2A and RH2B. [file 1471-2148-12-251-S3.pdf]

Additional file 3. Table of genotyping errors for 11 markers inferred from RAD-seq, including updated association values[c].

RAD-seq marker	LG	AA		AT		TT		ERROR (%)	*P*-value with opsin exp.
		RAD	SEQ	RAD	SEQ	RAD	SEQ		
74822	-	20	17	23	29	29	26	8.33	-
36342	-	19	16	40	45	12	10	12.68	-
56731	-	19	15	43	50	20	17	13.41	-
85240	-	19	19	38	44	22	16	11.39	-
4182	-	22	16	38	46	23	21	12.05	-
93007	-	41	36	15	29	12	3	20.59	-
59098	-	14	12	23	29	29	26	3.23	-
5611	5	13	8	21	29	20	17	14.81	< 0.0006
20245	10	20	15	31	36	25	25	7.89	< 2E-16
15377	14	19	19	31	34	27	24	9.09	-
28586	23	29	27	23	29	29	26	8.33	< 0.0003
Average	-	21.36	18.18	29.64	36.36	22.55	19.18	11.07	-

[c] P-value following ANOVA of cycle-sequencing genotypes and opsin expression of 5611 with *RH2B*; 20245 with *RH2A* and *LWS*; and 28586 with *SWS2B*, *SWS2A* and *RH2B*.
