# Supplementary material for: Evolution of cichlid vision via trans-regulatory divergence
Source: BMC Evol Biol. 2012 Dec 26;12:251. doi: 10.1186/1471-2148-12-251 (PMC3575402; doi:10.1186/1471-2148-12-251)
Supplement: Additional file 4 — Supplemental text discussing genotyping error and RAD-seq. [file 1471-2148-12-251-S4.pdf]

Supplemental discussion of genotyping error in RAD-seq

Error among genotypes inferred from RAD-seq could be caused by several intrinsic and introduced factors, including PCR-induced mutation, Illumina® sequencing bias, *de novo* sequence assembly or catalog construction, and genotyping cut-offs. Here, we review each of these possibilities and discuss their potential to explain the genotyping errors observed in this and other studies [1].

PCR Error. Dabney and Meyer [2] recently demonstrated that both the choice of PCR cycle number and *Taq* polymerase can influence the composition of next-generation sequencing libraries. Interestingly, these authors also found that the Phusion Taq recommended by Illumina and chosen by Etter et al. for RAD library construction [3] had one of the highest amplification biases of the polymerases tested. Although the RAD-seq protocol described in Etter et al. [3] calls for 18 PCR cycles with Phusion Taq and HF buffer, we amplified our libraries for 19 cycles. Thus, our choice of Phusion Taq and 19 cycles may have amplified the impact of PCR-induced mutations. However, error from PCR amplification should be < 1% per nucleotide, and these errors should occur randomly with respect to position and genotype. Thus, PCR-induced errors are not expected to consistently underestimate the number of heterozygous genotypes as observed in this study.

Another aspect of PCR-error, the systematic under-sampling of reads within reduced-representation libraries due to differences in PCR efficiency between SNPs and barcodes could also lead to genotyping error [3]. If the two alleles of a SNP are not sampled equally during library preparation, or the alleles/barcoes differ in their PCR efficiency, then the number of heterozygous genotypes at a given locus could be considerably reduced, possible leading the Stacks program to incorrectly infer a homozygous genotype. This type of bias in library

preparation and amplification could explain the reduced number of heterozygous loci found in our re-sequencing analysis (Additional file 3).

Illumina Sequencing Bias. G+C biases in amplified Illumina® sequence libraries are well documented, particularly at the 3' end of reads [4-6]. This bias could affect the composition of SNPs near the ends of untrimmed reads. Although we did not trim reads, we did filter them to ensure that overall sequence quality was high (Q20 across 90% of each read). We also required an individual to have a large number of reads (≥ 20x) at a locus before we inferred a genotype, and we chose conservative genotyping criteria that assigned as "missing" those genotypes with small but non-zero minor allele frequencies (0.02 – 0.08). These two changes should avoid spurious results due to sequencing error. But like Taq-error, Illumina biases are not expected to consistently underestimate the true number of heterozygous genotpes across multiple RAD-seq loci.

Sequence Assembly and Catalog Construction. Errors can also be introduced when reads are assembled into loci (called "stacks") or when orthologous stacks are combined into a common catalog of markers used to identify SNPs. When too few mismatches are allowed between reads, reads that contain alternate alleles in heterozygotes may be incorrectly assigned to different stacks, leading to an underestimate of heterozygous genotypes. In contrast, when too many mismatches are allowed, reads from different parts of the genome may be incorrectly combined into a common stack, increasing both the number of SNPs and heterozygous genotypes. The potential for stack assembly errors is considerable and should be proportional to the genetic distance between the parental species. In our case, different cichlid species are expected to show levels of genetic divergence comparable to many intraspecific populations, and we expect fewer than one polymorphism every 1 kb [7]; therefore, we chose to

allow only 1 mismatch per 100 bp read sequence. Assembly errors that are the result of allowing too few mismatches can be identified by the presence of too few heterozygotes and the presence of multiple loci that map to the same genomic position. Similarly, errors that are the result of allowing too many mismatches can be identified by stacks that contain multiple SNPs with a large number of heterozygous genotypes. We used a conservative set of assembly parameters that primarily generated stacks with only one SNP, and we filtered these SNPs by their adherence to Hardy-Weinberg equilibrium and genomic position. Although stacks in our P_0 may be under-assembled based on these criteria (see Additional file 1), this seems to be a result of the greater coverage and possibly greater sequencing error found in these individuals compared to the F_2. Increasing the number of mismatches allowed (*n* and *M* parameters in the Stacks script *denovo_map.pl* [8]) and using a random subset of the parental reads did generate fewer P_0 stacks, but did not change the number of stacks or reduce the percentage of genotyping errors found among the F_2.

Genotyping Cut-offs. Finally, the choice of genotyping parameters can also affect genotyping accuracy. We chose genotyping parameters that set all genotypes supported by less than 20 reads to "missing." We also set to "missing" genotypes where the frequency of the minor allele to the major one was between 0.02 and 0.08 (1/50 to 2/25). This range is larger than the default parameters in Stacks and should exclude most incorrect or ambiguous genotypes from our analysis. Manually inspecting questionable genotypes in the Stacks web-interface, particularly for the parental haplotypes, also reduced some errors.

Conclusions

Multiple factors may lead to genotyping errors in RAD-seq analyses; however, for some researchers, the ability to simultaneously identify and genotype hundreds of genome-wide

polymorphisms among non-model species may outweigh the risk of genotyping error. But these researchers should carefully consider ways to minimize error. Using fewer PCR cycles, different polymerases, and ensuring that there is sufficient coverage across each sample should help decrease the error rate of future RAD-seq analyses. And although some small amount of error is probably inevitable, these errors need not be considered fatal. The errors we identified did not inhibit our ability to construct linkage maps or successfully identify eQTL for cichlid opsin gene expression (Figure 2; Additional file 3).

References

1. Parnell NF, Hulsey CD, Streelman JT: **The genetic basis of a complex functional system.** *Evolution* 2012, **Epub ahead of print:** doi:10.1111/j.1558-5646.2012.01688.x.
2. Dabney J, Meyer M: **Length and GC-biases during sequencing library amplification: a comparison of various polymerase-buffer systems with ancient and modern DNA sequencing libraries.** *Biotechniques* 2012, **52:**87-94.
3. Etter PD, Bassham S, Hohenlohe PA, Johnson E, Cresko WA: **SNP Discovery and Genotyping for Evolutionary Genetics Using RAD Sequencing.** In *Molecular Methods for Evolutionary Genetics (Methods in Molecular Biology).* Edited by Orgogozo V, Rockman MV: Humana Press; 2011: 519
4. Aird D, Ross MG, Chen WS, Danielsson M, Fennell T, Russ C, Jaffe DB, Nusbaum C, Gnirke A: **Analyzing and minimizing PCR amplification bias in Illumina sequencing libraries.** *Genome Biol* 2011, **12:**R18.
5. Minoche AE, Dohm JC, Himmelbauer H: **Evaluation of genomic high-throughput sequencing data generated on Illumina HiSeq and Genome Analyzer systems.** *Genome Biol* 2011, **12:**R112.
6. Nakamura K, Oshima T, Morimoto T, Ikeda S, Yoshikawa H, Shiwa Y, Ishikawa S, Linak MC, Hirai A, Takahashi H, et al: **Sequence-specific error profile of Illumina sequencers.** *Nucleic Acids Res* 2011, **39:**e90.
7. Loh YH, Katz LS, Mims MC, Kocher TD, Yi SV, Streelman JT: **Comparative analysis reveals signatures of differentiation amid genomic polymorphism in Lake Malawi cichlids.** *Genome Biol* 2008, **9:**R113.
8. Catchen JM, Amores A, Hohenlohe P, Cresko W, Postlethwait JH: **Stacks: building and genotyping Loci de novo from short-read sequences.** *G3 (Bethesda)* 2011, **1:**171-182.
