# Supplementary figures and images for: Evolution of cichlid vision via trans-regulatory divergence
Source: BMC Evol Biol. 2012 Dec 26;12:251. doi: 10.1186/1471-2148-12-251 (PMC3575402; doi:10.1186/1471-2148-12-251)

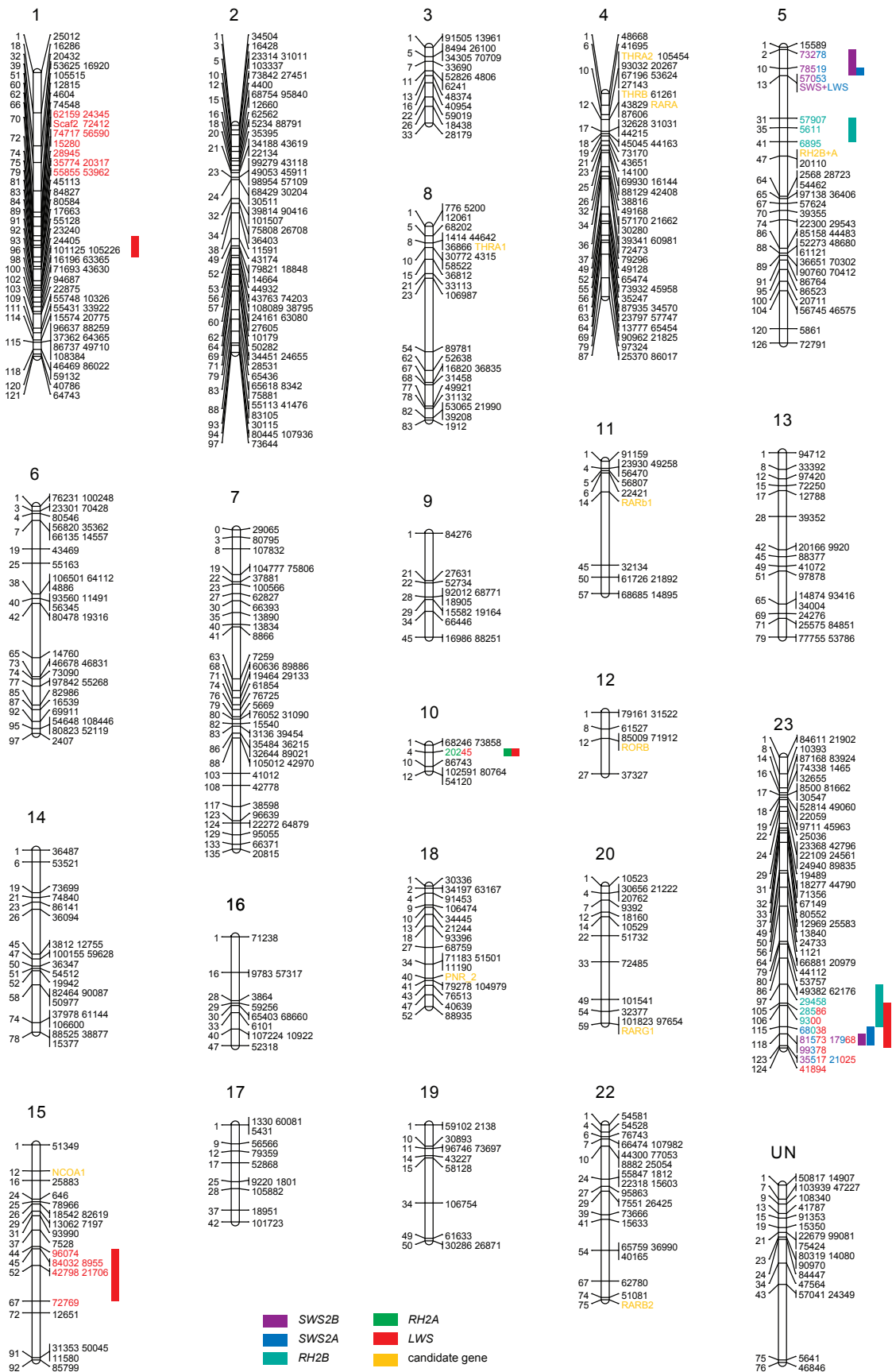

Supplement: Additional file 5 — Linkage map of the cichlid genome along with eQTL positions for cichlid opsin gene expression. [file 1471-2148-12-251-S5.pdf]

CIS

LG 5

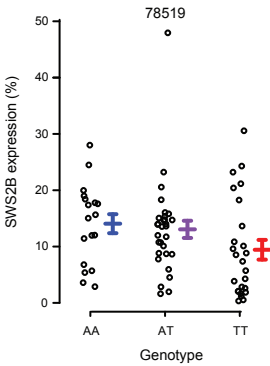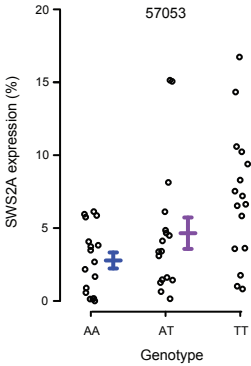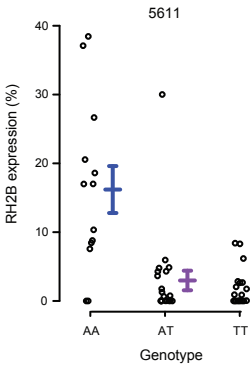

LG 1

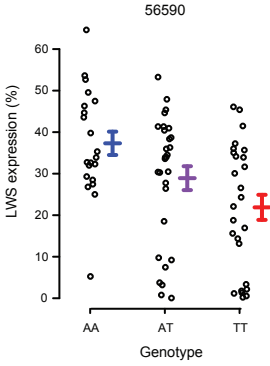

LG 10

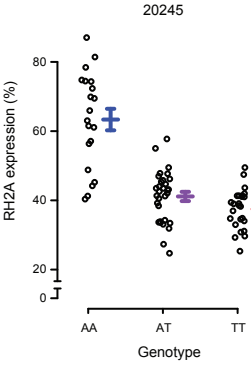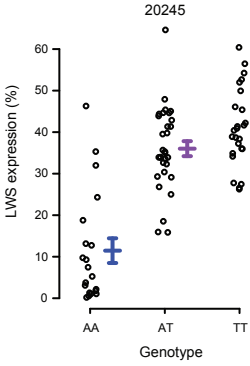

LG 15

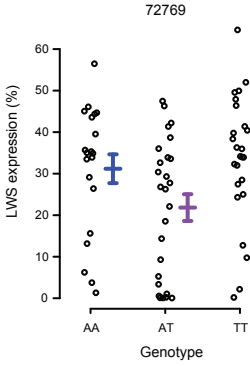

LG 23

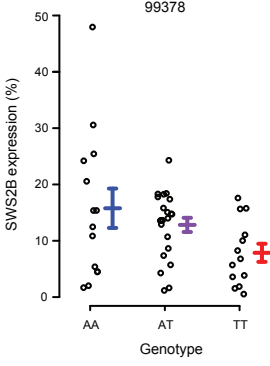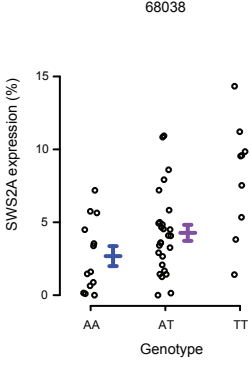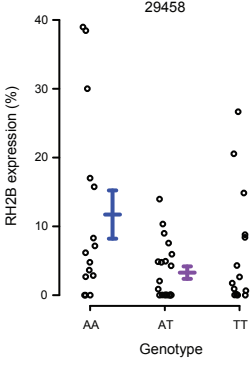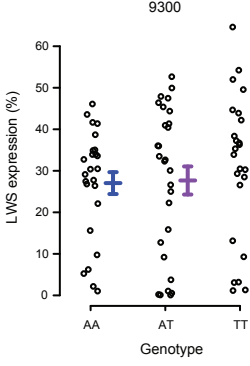

TRANS

Supplement: Additional file 7 — Distribution of opsin gene expression values among genotypic classes at eQTL loci. Genotypic classes include individuals that are homozygous for Aulonocara baenschi alleles (AA), homozygous for Tramitichromis intermedius alleles (TT), or heterozygous (AT). Marker loci are from the eQTL peaks indicated in Figure 2 and Table 2. Bars represent mean ± 1 SEM. The number of individuals varies among plots, though the average number of individuals is n = 62.60±4.06 SEM. [file 1471-2148-12-251-S7.pdf]
